# Fabrication of tin-filled carbon nanofibres by microwave plasma vapour deposition and their *in situ* heating observation by environmental transmission electron microscopy

**DOI:** 10.1186/1556-276X-8-302

**Published:** 2013-06-28

**Authors:** Tomoharu Tokunaga, Takumi Kanematsu, Takahumi Ito, Takahisa Ota, Yasuhiko Hayashi, Katsuhiro Sasaki, Takahisa Yamamoto

**Affiliations:** 1 Department of Quantum Engineering, Nagoya University, Furo-cho, Chikusa-ku, Nagoya, Aichi 464-8603, Japan; 2 Department of Frontier Materials, Nagoya Institute of Technology, Gokiso Showa, Gokiso-cho, Showa-ku, Nagoya, Aichi 466-8555, Japan; 3 Graduate School of Natural Science and Technology, Okayaha University, 3-1-1, Tsushima-naka, Kita-ku, Okayama-shi, Okayama 700-8530, Japan

**Keywords:** Nanofibre, Metal-filled CNF, Heating, *In situ* ETEM

## Abstract

Sn-filled carbon nanofibres (CNFs) are fabricated by microwave plasma chemical deposition. Scanning electron microscopy observations revealed the existence of a Sn island under the CNFs. The structure of the CNFs is investigated, and the behaviour of Sn in the internal space of CNFs is revealed by performing *in situ* heating observations by environmental transmission electron microscopy (ETEM). ETEM observations reveal that they have low-crystallized carbon wall and Sn occupies not only the CNF’s internal space but also its carbon wall. The Sn inside the CNF is completely covered by the carbon wall. Further, the *in situ* heating observations reveal that Sn within the internal space and the carbon wall of the CNFs diffused to the outside during heating. Moreover, it is found that higher membered carbon rings and defects in the graphite layer act as diffusion routes between disordered carbon layers.

## Background

Several methods for growing functionalized carbon nanotubes (CNTs) and carbon nanofibres (CNFs) have been proposed [1-4]. Further, methods for using the internal space of CNTs and CNFs have also been proposed. Some groups investigated methods for filling this internal space with metals during CNT and CNF growth [5-7]. Metal-filled CNFs (MFCNFs) are well-known carbon nanomaterials that can be easily fabricated by microwave plasma-enhanced chemical vapour deposition (MPCVD) with catalysts. During MPCVD, metal catalysts used in the fabrication of MFCNFs are introduced inside the MFCNFs. Various metals have been introduced into the internal space of MFCNFs, and the physical properties of these metals within the MFCNFs have been studied [5,8,9]. However, the behaviour of such metals inside CNFs and CNTs, especially under heating, has not been widely studied. In the present study, Sn-filled CNFs were fabricated by MPCVD and characterized by environmental transmission electron microscopy (ETEM). Moreover, *in situ* heating observations by ETEM were carried out to reveal the behaviour of Sn within the CNFs under heating.

## Methods

The Sn-filled CNFs were fabricated as follows: First, a thin Sn layer was fabricated on the surface of a 20 mm × 20 mm Si substrate with a natural oxide layer using a heating evaporation system. The evaporated substrate was transferred into an MPCVD chamber in air. The chamber was then evacuated to a pressure of 1 × 10^−5^ Pa. Next, hydrogen gas was introduced into the MPCVD chamber, and any remaining gas was purged from the chamber. The chamber pressure was kept at 20 Torr by introducing hydrogen gas at a flow rate of 50 sccm. The substrate was heated to 500°C and held at that temperature for 10 min under the hydrogen gas flow. Methane at 50 sccm and hydrogen at 50 sccm were introduced. The microwave plasma was then ignited, and a negative bias of 400 V was applied to the substrate, after which Sn-filled CNF growth began and continued for 10 min. The following conditions were maintained during the growth of the CNFs: a substrate temperature of 500°C, chamber pressure of 20 Torr, and microwave power of 700 W. After CNF growth, the microwave power was turned off, and the substrate temperature was gradually reduced under the hydrogen gas flow. When the substrate temperature reached approximately room temperature, the chamber pressure was brought up to atmospheric pressure by the introduction of nitrogen gas. Finally, the substrate was removed from the chamber. A commercial MPCVD system (Model AX5200, ASTeX, Cornes Technologies Limited, Minato-ku, Japan) was used for the fabrication of CNFs.

The Sn-filled CNFs grown on the Si substrate were characterized by ETEM (JEM-1000KRS, JEOL, Akishima-shi, Japan). They were collected from the substrate and deposited onto a metal grid thin foil with a carbon membrane using tweezers. The thin foil was then placed on a heated holder having a single-axis tilt mechanism (JEOL). The sample heating temperature was measured during the heating stage of the holder using a thermocouple placed directly in contact with the sample. The holder was inserted into the ETEM chamber, in which structural characterization, elemental analysis, and *in situ* heating observation by ETEM with electron energy loss spectroscopy (EELS) were performed. The sample heating temperature during the *in situ* observations was 400°C.

## Results and discussion

**Figure 1:**
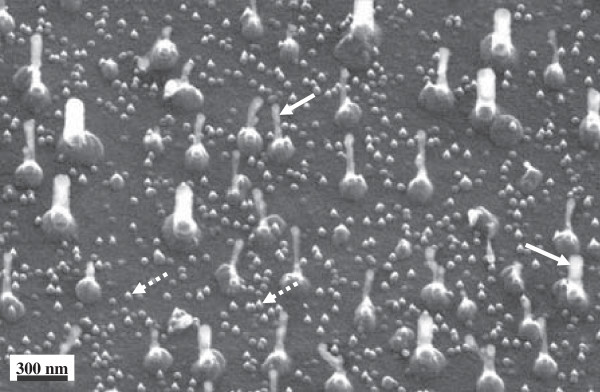
SEM image of as-grown Sn-filled CNFs on Si substrate.

Figure 1 shows a scanning electron microscopy (SEM) image of the as-grown Sn-filled CNFs on the Si substrate. The Sn-filled CNF yield was very small compared with that of CNFs grown using Fe, Co, and Ni as the catalyst [10-15]. Thin, long contrasts indicate CNFs, and bright areas, indicated by the solid white arrows, were confirmed around the central axis of the Sn-filled CNFs. The contrast in the SEM image originates from the emission of a second electron from a sample, and thus, bright contrasts indicate the existence of materials that differ from their surroundings. Further, these bright contrasts could be due to Sn, which is used as the catalyst, and/or Si, which is used as the substrate. Elemental analysis by EELS (described below) revealed that this bright contrast is due to Sn. Under the CNFs, islands, 150 nm in average diameter, necessarily existed. These islands possibly formed as particles owing to the shrinking of the evaporated Sn layer on the Si substrate when the substrate was annealed. Smaller diameter islands, indicated by broken white arrows in Figure 1, also formed along with the large islands. However, CNFs did not grow on the small islands, demonstrating that large-diameter islands are necessary for CNF growth. This article focuses on the structure, elemental analysis, and *in situ* observations of the CNFs, so the small-diameter islands are not described in detail. The CNFs were approximately 400 nm long and 30 to 100 nm in diameter.

**Figure 2:**
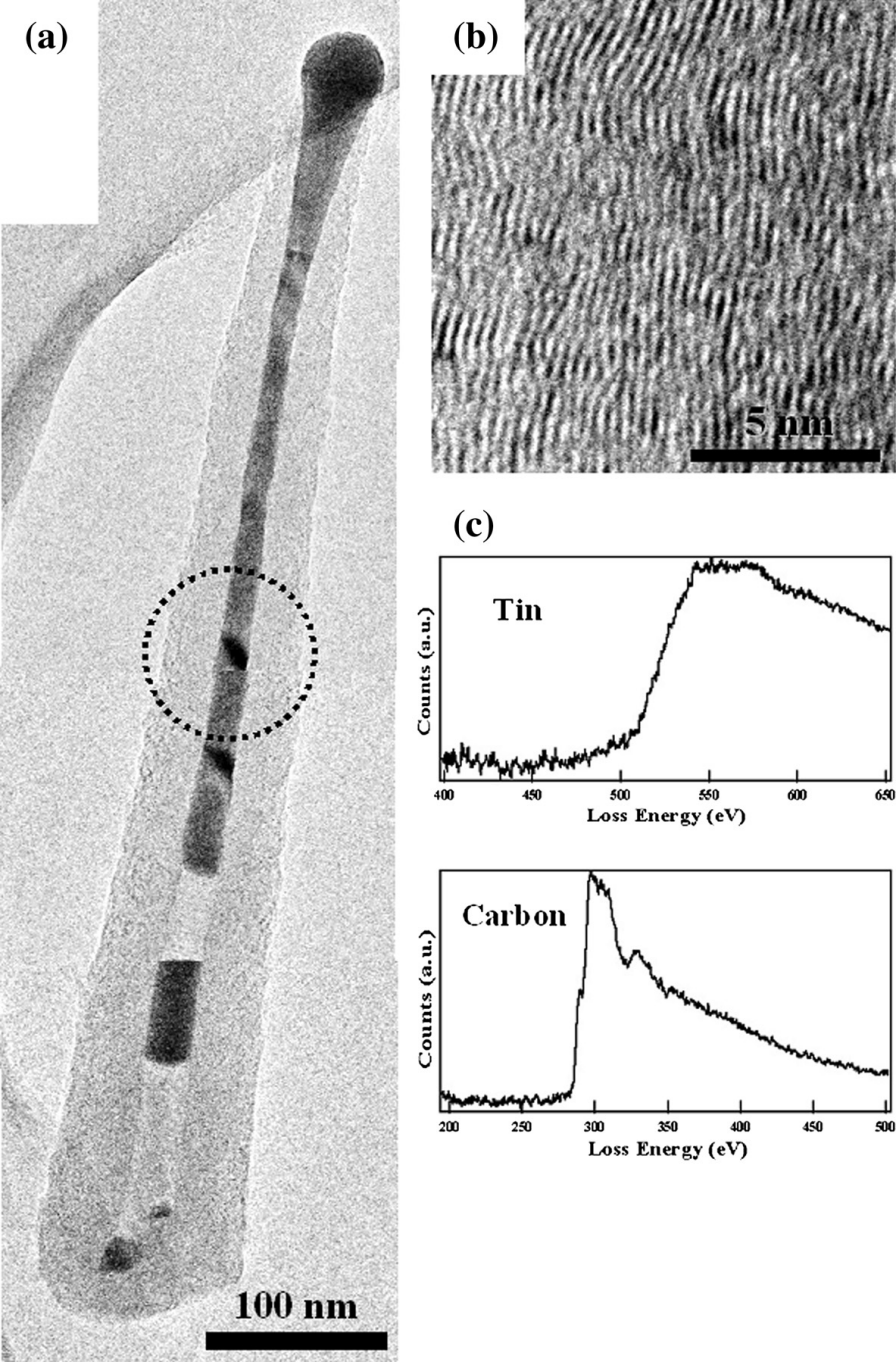
**TEM image of Sn-filled CNF, high-resolution TEM image of carbon wall, and EELS spectra.** (**a**) TEM image of Sn-filled CNF, (**b**) high-resolution TEM image of the carbon wall around the surface area of the Sn-filled CNF, and (**c**) EELS spectra from the area enclosed by a broken circle in Figure 2a.

**Figure 3:**
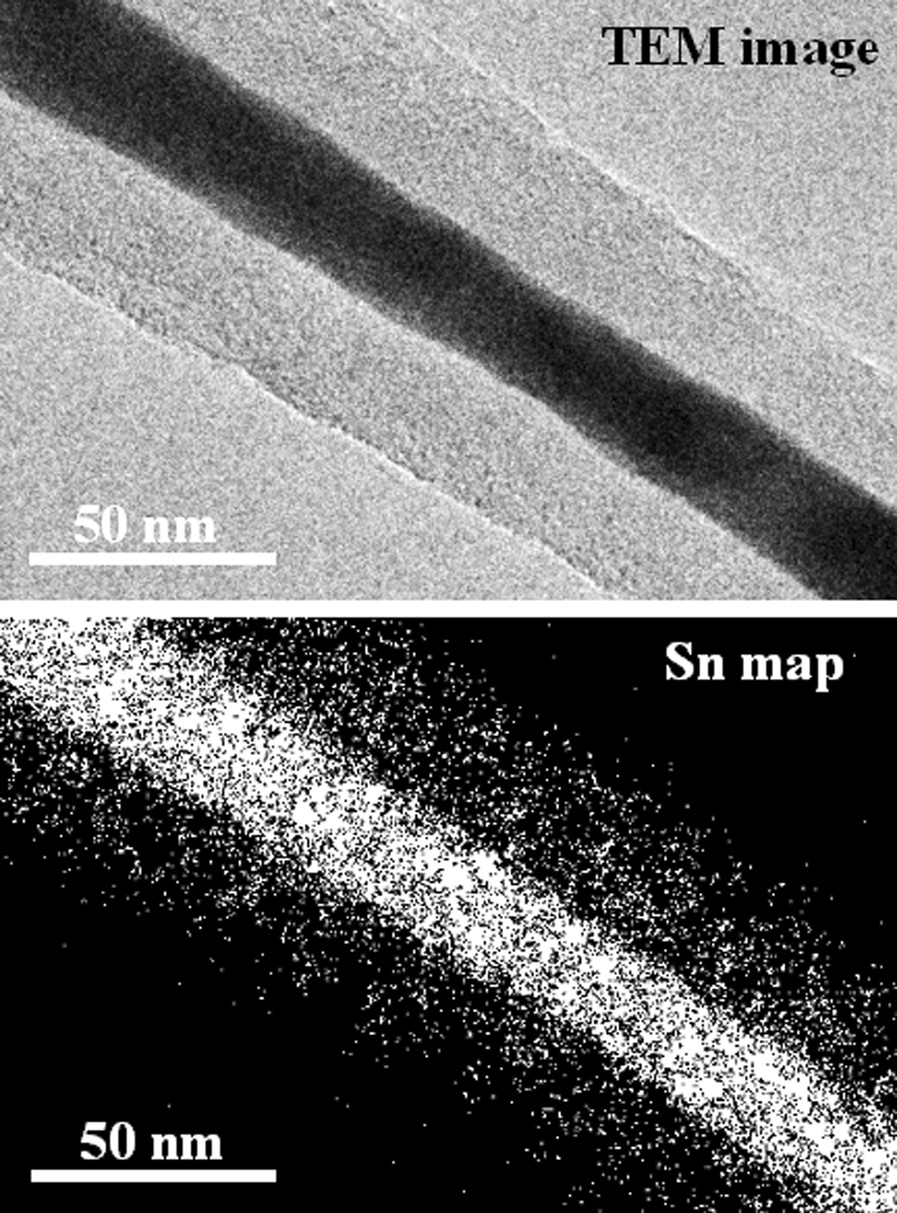
TEM image and Sn map of Sn-filled CNF.

Figure 2a shows a TEM image of a Sn-filled CNF collected from the Si substrate. The thin, long, rod-shaped contrast indicates the Sn-filled CNF, and the dark contrast seen at the central axis of the CNF confirms the existence of metal in its internal space. The carbon wall is identified by a slightly bright white area located around the dark contrast area. The dark contrast area fills the CNF. Figure 2b shows a high-resolution image of the carbon wall around the surface area in the Sn-filled CNF. Fringes at intervals of about 0.33 nm represent the distance between the graphite layers. These fringes are not straight but meandering and disjointed, indicating that the carbon wall of the CNF contains defects. EELS spectra for the elemental analysis were acquired from the CNF shown within the broken black circle in Figure 2a. The EELS spectra, shown in Figure 2c, confirm that the energy loss near the edge structure originated from Sn and C and that the CNF was made of Sn and C. Furthermore, Sn mapping of the Sn-filled CNF area shown in Figure 3 (top panel) was performed. The results of the Sn mapping, shown in Figure 3 (bottom panel), confirm the existence of Sn in the internal space of the CNF as well as in the carbon wall. The intensity of Sn in the carbon wall area was smaller than that around the central axis of the CNF, and this result showed that the amount of Sn in the carbon wall is seen to be lower than that around the central axis of the CNF. The above results reveal the successful growth of Sn-filled CNFs and the existence of Sn in the carbon walls of the grown CNFs.

Although many articles have reported the growth of metal-filled CNFs [12,15-17], the present study describes the first successful growth of Sn-filled CNFs on a Si substrate by MPCVD. Moreover, our results reveal the existence of Sn not only in the internal spaces of the Sn-filled CNFs but also in their carbon walls. The metal filling mechanism in the internal spaces of the CNFs was considered almost the same as that reported by Hayashi et al., in which metal is introduced to the internal space by a capillary effect during CNF growth [7]. Here, we discuss the reasons for the existence of Sn in the carbon wall. When the substrate was annealed, the Sn on the substrate formed particles. The plasma was then ignited, and the growth process began. The ions in the plasma collided with the surfaces of the Sn particles. Although these collisions increase the surface temperature of the particles, the exact temperature of the Sn particles was not determined. However, the surface temperature of the Sn particles is believed to have been approximately the same as the plasma temperature (several thousands of degrees Celsius [18]) because the substrate was covered completely by the plasma. The introduction of Sn into the carbon walls of the CNFs under these conditions could be explained by various phenomena. The first possibility is that Sn directly introduced into the carbon wall from under the CNF during the carbon wall owing to the solution of infinitesimal amounts of Sn in the carbon. However, the solid solution quantity of Sn in C is 0.002 at .% at several thousands of degrees Celsius [19]. The solution of Sn into the carbon wall could have dislocated the carbon wall during its formation, resulting in defects in the carbon wall. The second possibility is the diffusion of Sn present at the bottom of CNF as well as within the CNFs into the carbon wall. This diffusion of Sn could have occurred during plasma and substrate heating in the CNF growth process. The diffused Sn is considered to have remained in the carbon wall. The diffusion route of Sn in the carbon wall has been discussed in the paragraph describing the *in situ* heating observations. The third possibility is that Sn ions collided into the carbon wall. As mentioned above, the surface temperature of Sn particles on the substrate during MPCVD was extremely high. Previously reported MFCNFs had Fe, Co, Ni, or Cu only in their internal spaces [12,15-17], and these metals have high boiling points of 2,750°C, 2,900°C, 2,730°C, and 2,595°C [20], respectively. In contrast, the boiling point of Sn is about 2,270°C, which is lower than those of Fe, Co, Ni, and Cu. These values indicate that compared to these other metals, Sn is easier to evaporate at around the plasma temperature. This suggests that the Sn supplied in the plasma by Sn evaporation was ionized in the plasma, and the ionized Sn was attracted to the substrate by the negative bias, colliding with the CNFs growing on the substrate. The Sn was then deionized and remained in the carbon wall. When the ionized Sn collided with the CNFs, the fine carbon wall construction was possibly disturbed, damaging the carbon wall. There is also a possibility that Sn that was present on the substrate and sputtered by the bias-enhanced plasma collided with the CNFs. Sputtered metal typically exists as clusters in which some atoms aggregate. If clusters existed on and/or in the CNFs’ carbon walls, dark round contrasts would appear in TEM images. However, such dark contrasts do not appear in Figure 2a, so this possibility is low. These considerations leave us with the following three possibilities: Sn in the carbon wall was directly introduced to the carbon wall by the solution of Sn in carbon; Sn diffused into the carbon wall from beneath and within the CNF; and/or Sn on the substrate evaporated owing to heating by the plasma, and the evaporated Sn ionized in the plasma, collided with the CNFs, and diffused into the carbon wall.

**Figure 4:**
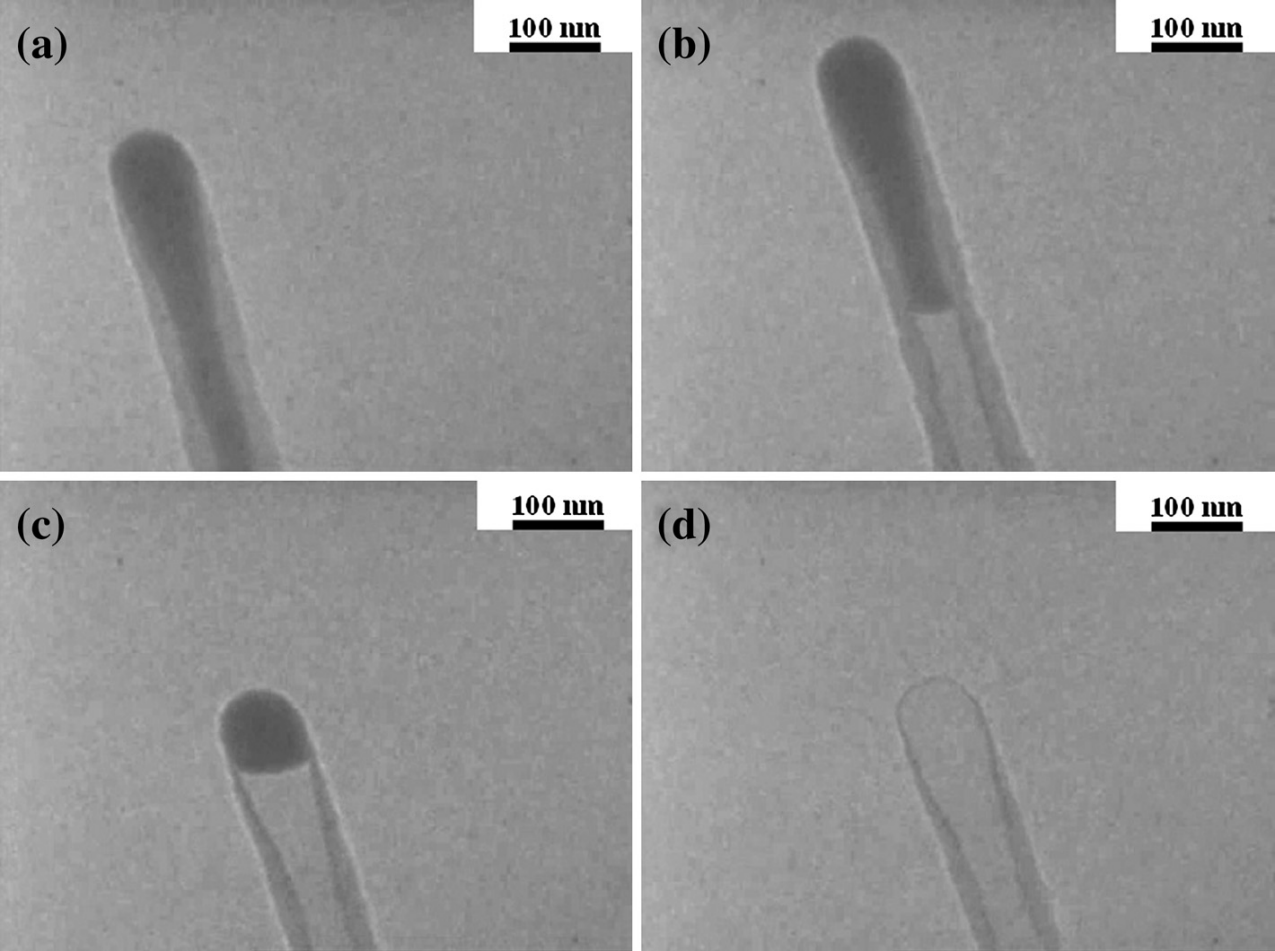
***In situ *heating TEM images of Sn-filled CNFs heated at 400°C.** (**a**) At the beginning of heating, (**b**) 1 min, (**c**) 3 min, and (**d**) 5 min.

**Figure 5:**
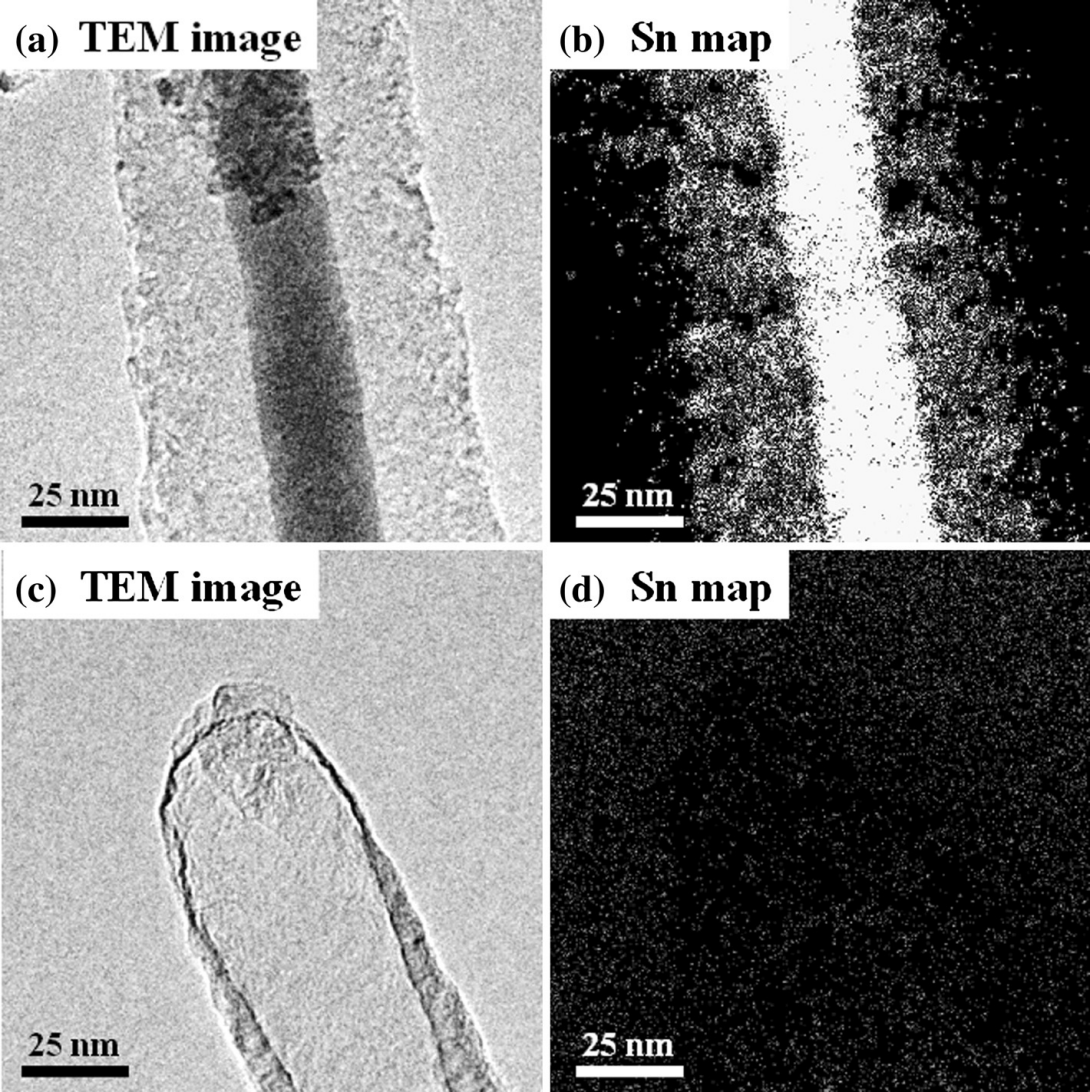
ETEM images and Sn maps of Sn-filled CNF (a, b) before and (c, d) after heating.

Next, we describe the *in situ* heating observations by ETEM. Figure 4 shows TEM images of the area around the tip of the Sn-filled CNF during heating at 400°C for several time periods. Figure 4a shows the beginning of heating, and the time increases from Figure 4b to Figure 4d. With increase in the heating time, the internal Sn gradually disappeared from the bottom of the CNF. Finally, Sn is completely eliminated from the CNF’s internal space and only remains in the carbon wall. This suggests that during heating, the Sn within the internal space of the CNF diffuses to the outside. Figure 5 shows Sn maps of the CNF during heating. The Sn in the carbon wall and the internal space observed is completely eliminated with continuous heating, as shown in the Sn map in Figure 5b, which was acquired from the CNF area shown in Figure 5a. This result demonstrates that Sn in the CNF’s carbon wall and internal space completely diffuses from inside the carbon wall and internal space to outside the CNF and may have evaporated.

These results clearly show that Sn can diffuse into the carbon wall of CNFs fabricated by MPCVD. The method of Sn diffusion into and out of the CNF is peculiar. It is certain that Sn diffused in the carbon wall because Sn was perfectly covered by the carbon wall (Figure 4). The carbon wall had a graphite structure (Figure 2b), and there are two possible routes for the Sn diffusion. One is the 0.33- to 0.34-nm gap between the graphite layers, and the other is a hole in the six-membered carbon ring, which is 0.14 nm on a side [21]. The maximum diameter of a six-membered ring is 0.28 nm, which is narrower than the distance between graphite layers. Hence, we speculate that Sn atoms diffuse preferentially in the space between the graphite layers. However, the carbon walls of our CNFs contain defects (Figure 2b), and hence, they exhibit a disordered structure similar to disordered graphite layers, higher membered carbon rings (e.g. seven- and eight-membered rings), and disjointedness in graphite layers. These structures are believed to function as the new third route for the Sn diffusion. Ng et al. suggested these three routes for the diffusion of Li ion into the carbon wall. In carbon rings, Li ions diffused more easily owing to defects such as those in carbon rings with more than six members [22]. In particular, carbon walls near the top of the CNFs have three-dimensionally curved walls such as those in fullerene, and hence, higher membered carbon rings exist at the top of the CNFs, leading to easy Sn diffusion there. As observed in Figure 4, Sn was eliminated from the top of the carbon wall of CNFs, which further suggests that Sn easily diffuses from the top of the CNFs. These *in situ* heating observation results provided us with remarkably important information that Sn can diffuse from within CNF carbon walls with defects to the outside of the CNF. This suggests that materials of approximately the same size or smaller than the Sn atoms can diffuse through a defective carbon wall.

It is expected that the Sn-filled CNFs fabricated by MPCVD in this study can be utilized for hydrogen storage. Metals used for hydrogen storage, such as Sn, are generally frazzled [23], and this shape is changed by hydrogen adsorption and desorption. This process degrades the hydrogen storage properties of the metals. In the Sn-filled CNFs fabricated in this study, Sn is covered by a carbon wall that may prevent Sn frazzling, thus helping Sn maintain its hydrogen storage properties. Thus, the Sn-filled CNFs can likely be used as a hydrogen storage material.

## Conclusions

We carried out structural analysis and *in situ* heating observations of Sn-filled CNFs grown by MPCVD. Sn was found to exist in the internal spaces as well as the carbon walls of the CNFs. Three possible mechanisms for the introduction of Sn into the carbon wall were discussed. The first possibility is that Sn was introduced directly from the Sn particles on the substrate during CNF growth. The second is that Sn diffused from the Sn beneath and within the CNF. The third is that Sn evaporated into plasma by the high plasma temperature collided with the CNF wall and was introduced into the carbon wall by negative bias. Moreover, by observing the heating of Sn-filled CNFs, we confirmed that Sn in the internal space and in the carbon wall of the CNF diffused to the outside through the carbon wall. The Sn is considered to pass through the space between disordered carbon layers, higher membered carbon rings, and defects in the graphite layer.

## Competing interests

The authors declare that they have no competing interests.

## Authors’ contributions

TT planned the overview of this study, designed and carried out the ETEM observation, and drafted the manuscript. TK operated and analysed the EELS mapping by ETEM. TI carried out the design of sample fabrication set-up. TO carried out the fabrication sample. YH maintained the fabrication MPCVD setup. KS maintained and set up the ETEM. TY designed the ETEM observation condition. All authors read and approved the final manuscript.
